# Systems pharmacology of adiposity reveals inhibition of EP300 as a common therapeutic mechanism of caloric restriction and resveratrol for obesity

**DOI:** 10.3389/fphar.2015.00199

**Published:** 2015-09-15

**Authors:** Yuhei Nishimura, Shota Sasagawa, Michiko Ariyoshi, Sayuri Ichikawa, Yasuhito Shimada, Koki Kawaguchi, Reiko Kawase, Reiko Yamamoto, Takuma Uehara, Takaaki Yanai, Ryoji Takata, Toshio Tanaka

**Affiliations:** ^1^Department of Molecular and Cellular Pharmacology, Pharmacogenomics and Pharmacoinformatics, Mie University Graduate School of MedicineTsu, Japan; ^2^Mie University Medical Zebrafish Research CenterTsu, Japan; ^3^Department of Systems Pharmacology, Mie University Graduate School of MedicineTsu, Japan; ^4^Department of Omics Medicine, Mie University Industrial Technology Innovation InstituteTsu, Japan; ^5^Department of Bioinformatics, Mie University Life Science Research CenterTsu, Japan; ^6^Product Development Research Institute, Mercian CorporationFujisawa, Japan

**Keywords:** resveratrol, caloric restriction, obesity, adipose tissue, ep300, zebrafish, comparative transcriptome, systems pharmacology

## Abstract

Both caloric restriction (CR) and resveratrol (RSV) have beneficial effects on obesity. However, the biochemical pathways that mediate these beneficial effects might be complex and interconnected and have not been fully elucidated. To reveal the common therapeutic mechanism of CR and RSV, we performed a comparative transcriptome analysis of adipose tissues from diet-induced obese (DIO) zebrafish and obese humans. We identified nine genes in DIO zebrafish and seven genes in obese humans whose expressions were regulated by CR and RSV. Although the gene lists did not overlap except for one gene, the gene ontologies enriched in the gene lists were highly overlapped, and included genes involved in adipocyte differentiation, lipid storage and lipid metabolism. Bioinformatic analysis of *cis*-regulatory sequences of these genes revealed that their transcriptional regulators also overlapped, including EP300, HDAC2, CEBPB, CEBPD, FOXA1, and FOXA2. We also identified 15 and 46 genes that were dysregulated in the adipose tissue of DIO zebrafish and obese humans, respectively. Bioinformatics analysis identified EP300, HDAC2, and CEBPB as common transcriptional regulators for these genes. EP300 is a histone and lysyl acetyltransferase that modulates the function of histone and various proteins including CEBPB, CEBPD, FOXA1, and FOXA2. We demonstrated that adiposity in larval zebrafish was significantly reduced by C646, an inhibitor of EP300 that antagonizes acetyl-CoA. The reduction of adiposity by C646 was not significantly different from that induced by RSV or co-treatment of C646 and RSV. These results indicate that the inhibition of EP300 might be a common therapeutic mechanism between CR and RSV in adipose tissues of obese individuals.

## Introduction

According to the World Health Organization, an estimated 310 million people worldwide are obese (Bessesen, 2008). Such estimates are particularly alarming given the strong association between obesity and various adverse health consequences, including atherosclerosis, hypertension, type 2 diabetes and certain types of cancer (Bessesen, 2008). CR can alleviate these deleterious conditions in obesity (Guarente, 2013). However, eating less for the sake of creating a desirable metabolic profile is unlikely to gain widespread compliance (Timmers et al., 2011). Accordingly, there has been an increasing interest in identifying compounds that elicit the beneficial effects of CR without requiring reduced calorie intake.

Resveratrol is thought to mimic the effects of CR in obesity (Guarente, 2013). In mice on a high-fat diet, RSV diminished total body fat content and decreased depots of epididymal, inguinal and retroperitoneal white adipose tissue (Lagouge et al., 2006). In obese Zucker rats, the administration of RSV resulted in a significant reduction in plasma TGs, free fatty acids, cholesterol, and liver TGs when compared with untreated obese Zucker rats (Rivera et al., 2009).

The mechanism of how RSV exerts these favorable effects was proposed to be related to the induction of genes encoding oxidative phosphorylation and mitochondrial biogenesis molecules (Chung et al., 2012; de Ligt et al., 2015). Numerous data indicate that the activation of NAD^+^-dependent protein deacetylase, SIRT1, is pivotal for the beneficial effect of RSV (Chung et al., 2012; de Ligt et al., 2015). SIRT1 catalyzes, among others, deacetylation and the activation of peroxisome proliferator gamma coactivator-1α, a cofactor in mitochondrial biogenesis (Chung et al., 2012; de Ligt et al., 2015). However, the biochemical pathways proposed to mediate the beneficial effects of RSV are highly interconnected (Chung et al., 2012), suggesting that systems pharmacology might be required to fully elucidate the therapeutic mechanism of RSV in obesity.

It was demonstrated that CR increases the expression of SIRT1 protein (Cohen et al., 2004) and that the beneficial effects of CR are mediated via SIRT1 (Picard et al., 2004). However, the general up-regulation of Sirt1 expression was challenged by a study showing CR-regulated Sirt1 expression was tissue specific in mice (Chen et al., 2008). Moreover, SIRT1-independent effects of CR were also reported (Kaeberlein and Powers, 2007). These findings suggest there common therapeutic mechanisms between CR and RSV might be both dependent and independent of SIRT1.

To identify the common therapeutic mechanism between CR and RSV, we performed comparative transcriptome analysis of adipose tissues from DIO zebrafish and obese humans with or without CR or RSV. Combined with bioinformatics analysis, we identified EP300, a histone and lysyl acetyltransferase, as a master regulator for genes dysregulated in obesity and normalized by CR and RSV. We also demonstrated that the inhibition of EP300 by a competitive inhibitor of acetyl-CoA reduced the adiposity of larval zebrafish. These results suggest that the inhibition of EP300 might be a common therapeutic mechanism of CR and RSV on adiposity in obesity.

## Materials and Methods

### Ethics Statement

Mie University Institutional Animal Care and Use Committee guidelines state that no approval is required for experiments using zebrafish. However, animal experiments described in this manuscript conform to the ethical guidelines established by the Institutional Animal Care and Use Committee at Mie University.

### Compounds and Reagents

Resveratrol and C646 were purchased from Sigma (St. Louis, MO, USA). Nile Red was purchased from Tokyo Kasei (Tokyo, Japan); 2-phenoxyethanol and TG L-type assay were purchased from Wako (Tokyo, Japan).

### Zebrafish Husbandry

Zebrafish were maintained according to the methods described by Westerfield (2007) with some modification. Briefly, zebrafish were raised at 28.5 ± 0.5°C with a 14 h/10 h light/dark cycle. Embryos were obtained via natural mating and cultured in 0.3 × Danieau solution [19.3 mM NaCl, 0.23 mM KCl, 0.13 mM MgSO_4_, 0.2 mM Ca(NO_3_)_2_, 1.7 mM HEPES, pH 7.2]. To induce the adult DIO model, we used a transparent zebrafish mutant line *MieKomachi 001* (*MK001*) that was created by crossing *nacre* and *rose.* For the experiments using larva, we used an albino zebrafish line that was obtained from the Max Planck Institute for Developmental Biology (Tübingen, Germany).

### Adult DIO Zebrafish Model

Female zebrafish at 4 mpf were assigned into three dietary groups: OF, OF + RSV, and control, with five fish per 2-L tank. Zebrafish in the OF and OF + RSV groups were fed three times per day with freshly hatched Artemia (corresponding to 60 mg cysts/fish/day). Zebrafish in the control group were fed freshly hatched *Artemia* (corresponding to 5 mg cysts/fish/day) once per day. Zebrafish in the OF + RSV groups were fed with gluten containing RSV (corresponding to 20 μg /fish/day) at 20 min before feeding *Artemia* in the morning. Zebrafish in the OF and control groups were fed with gluten without RSV at 20 min before feeding *Artemia* in the morning. Gluten with or without RSV were prepared as previously described (Zang et al., 2011). The body weight and length of zebrafish were measured weekly throughout the study. Zebrafish length was measured from the head to the end of the body. Blood collection and TG measurement in the plasma were performed as described previously (Oka et al., 2010). Staining visceral adipose tissues was performed as previously described (Oka et al., 2010) except for the concentration of Nile Red (1 μg/ml) and the staining time (30 min). After staining, the visceral adipose tissues were observed with a fluorescence microscope (MZ 16F, Leica, Tokyo, Japan) using a GFP2 filter (Leica). The fluorescent intensity of Nile Red was calculated using Volocity 3D Image Analysis Software (Perkin-Elmer, Cambridge, MA, USA).

### Assessment of Adiposity in Larval Zebrafish

Zebrafish at three dpf were treated with 2 μM C646, 200 μM RSV or 2 μM C646 and 200 μM RSV for 48 h. After the treatment, zebrafish were stained with Nile Red (5 ng/ml) for 30 min. After staining, zebrafish were rinsed in 0.3 × Danieau solution and anesthetized with 2-phenoxyethanol (500 ppm). *In vivo* imaging of the zebrafish was performed using a fluorescence microscope (SMZ25, Nikon, Tokyo, Japan) with a GFP-L filter (Nikon). The area stained with Nile Red in the abdomen was measured using Volocity (Perkin-Elmer).

### Transcriptome Analysis of Visceral Adipose Tissues of Female DIO Zebrafish

The visceral adipose tissue of female DIO zebrafish overfed Artemia for 1 week (OF 1 w) or 5 weeks (OF 5 w), or overfed Artemia for 5 weeks with RSV (OF + RSV 5 w), were stained with Nile Red and collected by surgical extraction under a fluorescence microscope. The visceral adipose tissue was stored in RNA-later (Applied Biosystems, Foster City, CA, USA). Total RNA was then extracted using an RNeasy Plus Micro Kit (Qiagen, Valencia, CA, USA), qualified by an Agilent Bioanalyzer 2100 (Agilent, Santa Clara, CA, USA) and quantified using a spectrophotometer (NanoDrop ND-100, Wilmington, DE, USA). Fifty nanograms of total RNA from each visceral adipose tissue depot were converted into labeled cRNA using the Low RNA Input Fluorescent Linear Amplification Kit (Agilent). Cy3-labeled cRNA (860 ng) was hybridized to Agilent Zebrafish Whole Genome Oligo Microarrays (G2519F) according to the manufacturer’s protocol. The hybridized microarrays were scanned (Agilent G2565BA) and analyzed using Feature Extraction software (Agilent). The data were normalized using Limma (Ritchie et al., 2015), a package in Bioconductor. Probes that passed four criteria (gIsSaturated, gIsFeatNonUnifOL, gIsPosAndSignif, gIsWellAboveBG) across the dataset were used for further analysis. RankProd analysis (Hong et al., 2006) was performed to identify differentially expressed genes between two groups by calculating the false discovery rate (FDR). Differentially expressed genes (FDR < 30%) were then converted to human orthologs using the Life Science Knowledge Bank (World Fusion, Tokyo, Japan). The gene symbols of human orthologs were used for functional analysis. The gene symbols of human orthologs were used for functional analysis. The microarray data has been deposited to GEO as GSE70281.

### Transcriptome Analysis of Adipose Tissue in Male DIO Zebrafish and Obese Humans

We downloaded transcriptome data from the GEO analyzing visceral adipose tissues of male DIO zebrafish with and without CR (GSE18566; Oka et al., 2010), subcutaneous adipose tissues of obese humans with and without CR (GSE35710; Nookaew et al., 2013), subcutaneous adipose tissues of obese human males with and without RSV (GSE42432; Konings et al., 2014), subcutaneous adipose tissue of obese human females (GSE44000; Deng et al., 2013), and subcutaneous adipose tissue of obese human males (GSE29718; Tam et al., 2011).

For the Agilent array (GSE18566 and GSE44000), transcriptome data were normalized using Limma (Ritchie et al., 2015) and the probes that passed four criteria (gIsSaturated, gIsFeatNonUnifOL, gIsPosAndSignif, gIsWellAboveBG) across the dataset were used for further analysis. For the affymetrix array, the transcriptome data were normalized using “affy” (Bolstad et al., 2003) for GSE35710 or “oligo” (Carvalho et al., 2007) for GSE29718 and GSE42432. Data normalized by “affy” were filtered based on the presence-absence call and probes that were present or marginal across the dataset were used for further analysis. Data normalized by “oligo” were filtered based on the normalized signal and probes with a normalized signal >3 across the dataset were used for further analysis. RankProd analysis (Hong et al., 2006) was performed to identify differentially expressed genes between two groups by calculating the FDR. FDR30% was used as the threshold. For transcriptome analysis of zebrafish, differentially expressed genes were converted to human orthologs using the Life Science Knowledge Bank (World Fusion, Tokyo, Japan).

### Identification of Transcriptional Regulators using iRegulon

iRegulon (Janky et al., 2014) exploits the fact that genes that are co-regulated by the same TF commonly share binding sites for the TF and uses gene sets derived from ENCODE ChIP-seq data. We used iRegulon as an application in Cytoscape (Shannon et al., 2003). The lists of differentially expressed genes shown in **Supplementary Table S1** were subjected to iRegulon and used to predict their transcriptional regulators using the default setting. The predicted transcriptional regulators with normalized enrichment scores (NES) >3 are shown in **Supplementary Table S4** (Sheets 1–4) and used for further analysis.

### Identification of Transcriptional Regulators using Pathway Studio

Pathway Studio (Nikitin et al., 2003) uses gene sets derived from natural language processing based text mining of published literature, including a gene set for transcriptional regulators, composed of genes whose promoters the transcriptional regulator bound to and genes whose expression were regulated by the transcriptional regulator. The lists of differentially expressed genes shown in **Supplementary Table S1** were subjected to Pathway Studio and used to predict their transcriptional regulators using the subnetwork enrichment analysis for “expression target”. The predicted transcriptional regulators with *p* < 5.0 × 10^-3^ are shown in **Supplementary Table S5** (Sheets 1–4) and were used for further analysis.

### Identification of Cell Processes Enriched in a Gene List using Pathway Studio

The lists of differentially expressed genes were subjected to Pathway Studio and used to predict cell processes related to the lists using the subnetwork enrichment analysis with *p* < 5.0 × 10^-3^ as the threshold.

### *K*-Means Clustering

*K*-means clustering was performed using MultiExperiment Viewer v4.8 (Howe et al., 2011).

### Statistical Analysis

Statistical analysis (analysis of variance followed by Tukey’s multiple comparisons test) was performed using Prism 6.0 (GraphPad Software, San Diego, CA, USA).

## Results

### Resveratrol Reduces Plasma Triglyceride and Visceral Fat in Diet-Induced Obese Zebrafish

We previously demonstrated that CR after OF significantly lowered both plasma TG and visceral adiposity in zebrafish (Oka et al., 2010). In this study, we first examined whether RSV could also exert these effects in the DIO zebrafish model. Adult female zebrafish were overfed with freshly hatched nauplii of *Artemia* that have a relatively high fat content compared with flake foods (Oka et al., 2010) with and without RSV treatment (20 μg/day, 40 mg/kg body weight/day in 0.5 g zebrafish). The BMI of OF zebrafish was significantly increased compared with the BMI of control zebrafish after 1 week (**Supplementary Figure S1A**). The BMI of zebrafish overfed with *Artemia* and treated with RSV (OF + RSV) was also significantly increased compared with the BMI of control zebrafish. However, the BMI of OF + RSV zebrafish was not significantly different from that of OF zebrafish. We then examined the effects of RSV on plasma TG. As shown in **Supplementary Figure S1B**, plasma TG in OF zebrafish was significantly increased compared with that of control zebrafish. In contrast, plasma TG levels in OF + RSV zebrafish were significantly lower than in OF zebrafish. We also examined the effects of RSV on visceral adiposity in DIO zebrafish, which can be visualized by Nile Red staining (Oka et al., 2010). As shown in **Supplementary Figure S1C**, the fluorescent intensity of Nile Red in OF zebrafish was significantly higher than in control zebrafish. The fluorescent intensity of Nile Red in OF + RSV zebrafish was significantly lower than in OF zebrafish. These results suggest that RSV can reduce plasma TG and adiposity in DIO zebrafish, consistent with previous reports in mammalian obesity (Rocha et al., 2009; Timmers et al., 2011).

### Identification of Transcriptional Regulators for Genes Commonly Regulated by RSV and CR in DIO Zebrafish

**FIGURE 1:**
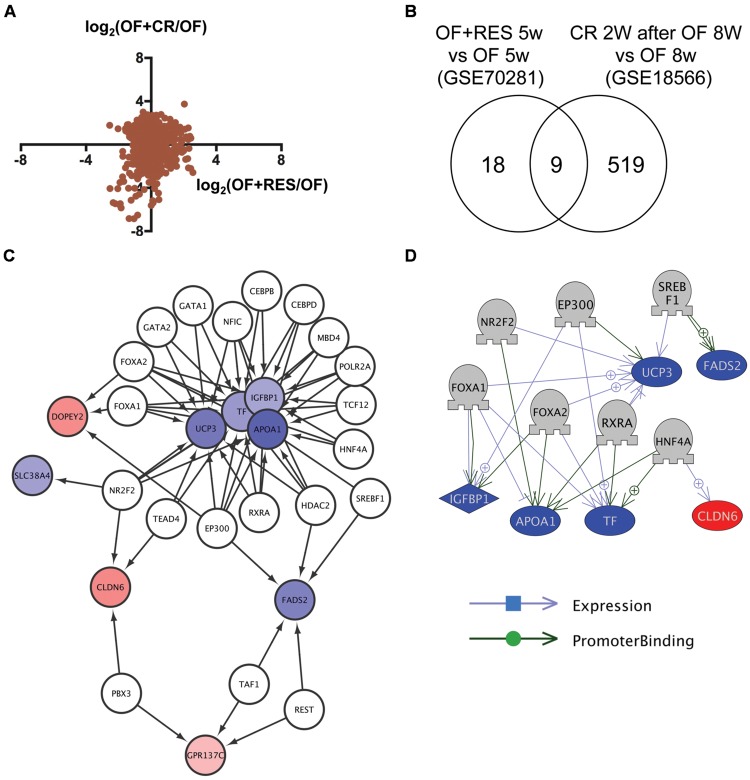
**Identification of transcriptional regulators for genes with changed expression levels common to RSV and CR in DIO zebrafish. (A)** Correlation of gene expression changes induced by RSV and CR in DIO zebrafish. **(B)** Venn diagram of the numbers of DEG by RSV and CR in DIO zebrafish. **(C)** A regulatory network identified by iRegulon for the nine genes common between the RSV and CR groups. Genes shown in blue and red are decreased and increased, respectively, by RSV or CR. **(D)** A regulatory network identified by Pathway Studio for seven regulators and their six target genes. Genes shown in blue and red indicate decreased and increased expression, respectively, by RSV or CR.

To identify common mechanisms of action between RSV and CR, we compared transcriptome data of visceral adipose tissue in DIO zebrafish with RSV or CR (Oka et al., 2010). Although the correlation of change in gene expression between RSV and CR was low (**Figure 1A**), we identified nine genes in common (**Figure 1B** and **Supplementary Table S1**, Sheet 1) from 27 and 528 genes whose expression were changed by RSV (**Supplementary Table S2**, Sheet 1) and CR (**Supplementary Table S2**, Sheet 2), respectively. Gene ontology analysis revealed that 56 cell processes, including lipid peroxidation, lipid storage, and energy homeostasis, were enriched in the nine identified genes (**Supplementary Table S3**, Sheet 1). To reveal the molecular mechanism involved in the regulation of the expression of the nine common genes, we used iRegulon (Janky et al., 2014) to identify master regulators of the co-expressed genes based on the genome-wide ENCODE ChIP-seq data. iRegulon identified 20 transcriptional regulators for the nine genes with a NES >3 (**Supplementary Table S4**, Sheet 1). The network between these transcriptional regulators and their target genes is shown in **Figure 1C**. To validate the network identified by iRegulon, we used Pathway Studio (Nikitin et al., 2003) to identify the master regulators of co-expressed genes using natural language processing based text mining. Pathway Studio identified 50 regulators for the nine common genes (**Supplementary Table S5**, Sheet 1). Seven regulators were overlapped between iRegulon and Pathway Studio data. The network between the seven regulators and their target genes identified by Pathway Studio is shown in **Figure 1D**. The overlapping network correlated well with the data identified by iRegulon. For example, Pathway Studio identified FOXA1/FOXA2 as positive regulators of *IGFBP1, TF*, and *UCP3*. iRegulon also identified these three genes as targets of FOXA1/FOXA2 **Supplementary Table S4**, Sheet 1).

### Identification of Transcriptional Regulators for Genes Commonly Regulated by RSV and CR in Obese Humans

**FIGURE 2:**
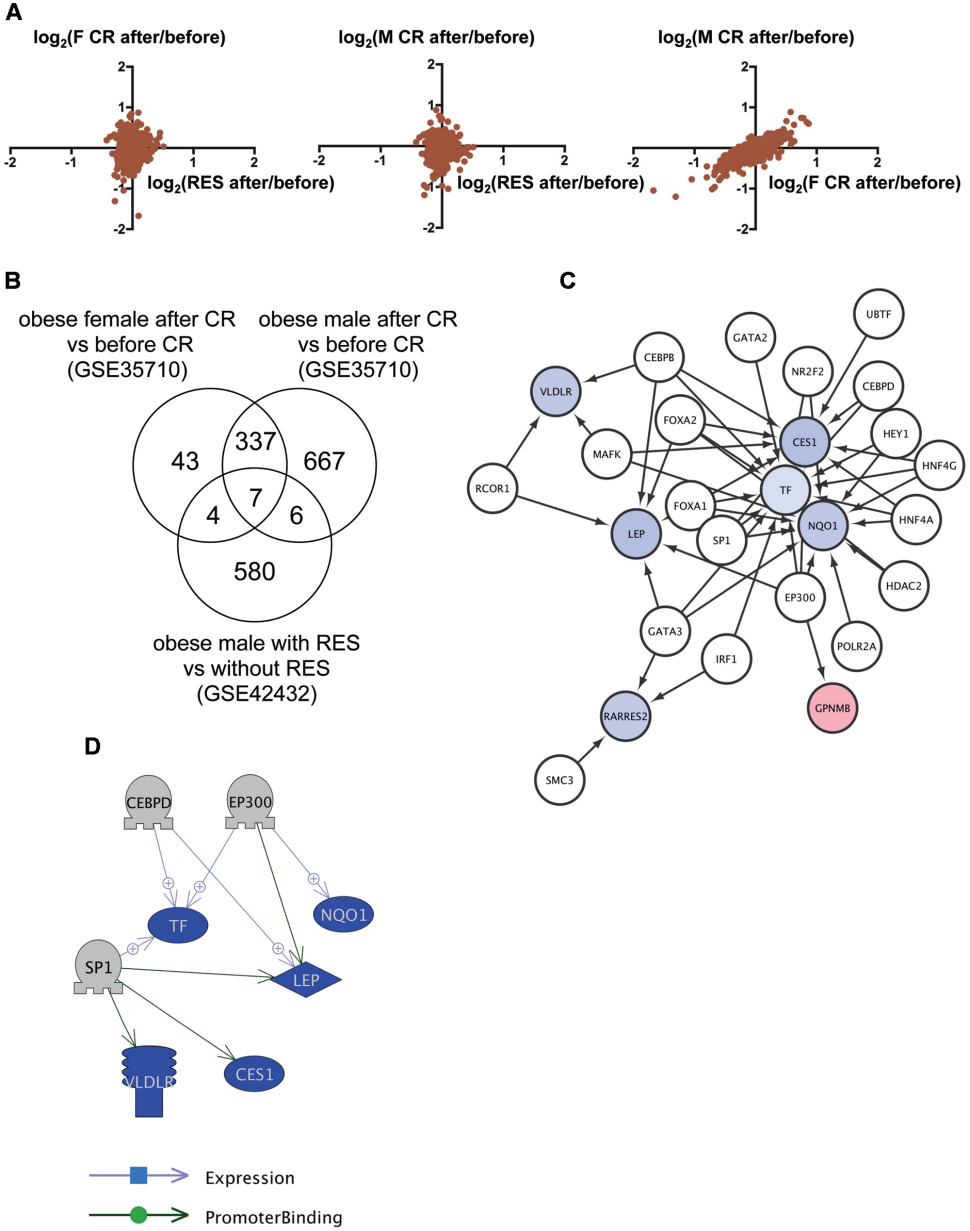
**Identification of the regulatory network of genes with changed expression levels common to RSV and CR in obese humans. (A)** Correlation of gene expression changes by RSV in obese males and CR in obese females (left) or males (middle) and CR in obese females and males (right). **(B)** Venn diagram of the numbers of DEG by RSV in obese males, or CR in obese females or males. **(C)** A regulatory network identified by iRegulon for the seven common genes among RSV in obese males, and CR in obese females and males. Genes shown in blue and red indicate decreased and increased expression, respectively, by RSV or CR. **(D)** A regulatory network identified by Pathway Studio between three regulators and their five target genes. Genes shown in blue and red indicate decreased and increased expression, respectively, by RSV or CR.

We then applied this analysis to determine the common mechanism of action of RSV and CR in adipose tissues of obese humans. We analyzed the transcriptome data of subcutaneous adipose tissues from obese humans with and without RSV (Konings et al., 2014) or CR (Nookaew et al., 2013) deposited to GEO as GSE42432 and GSE35710, respectively. GSE42432 contained data from males only, whereas GSE35710 contained data from both females and males. The correlation of gene expression between RSV and CR was low (**Figure 2A**, left and middle), which is consistent with the result in DIO zebrafish (**Figure 1A**), while the correlation between female and male CR was high (*r* = 0.87, *p* < 1 × 10^-4^, **Figure 2A**, right). We identified seven genes in common (**Figure 2B**, **Supplementary Table S1**, Sheet 2) among 597 genes with altered expression by RSV (**Supplementary Table S2**, Sheet 3), and 391 and 1,017 genes with altered expression by CR in females and males, respectively (**Supplementary Table S2**, Sheets 4 and 5). Gene ontology analysis revealed that 177 cell processes, including lipid metabolism, lipid degradation, and mitochondrial depolarization were enriched in the seven genes (**Supplementary Table S3**, Sheet 2). Although only one gene (transferrin) was overlapped between the nine and seven common genes in zebrafish and humans, respectively (**Supplementary Table S1**, Sheets 1 and 2), 36 gene ontologies were overlapped between the 56 and 177 gene ontologies in zebrafish and humans, respectively (**Supplementary Table S3**, Sheets 1 and 2). iRegulon identified 19 transcriptional regulators for the seven genes (**Supplementary Table S4**, Sheet 2). The network between these transcriptional regulator and their targets is shown in **Figure 2C**. Pathway Studio identified 34 regulators for the seven common genes (**Supplementary Table S5**, Sheet 2). Three regulators were overlapped between data from the iRegulon and Pathway Studio analyses. The network between the three regulators and their target genes identified by Pathway Studio is shown in **Figure 2D**. The overlapping network correlated well with the data identified by iRegulon. For example, Pathway Studio identified EP300 as a positive regulator of *LEP, TF*, and *NQO1*. iRegulon also identified these three genes as targets of EP300 (**Supplementary Table S4**, Sheet 2).

### Identification of Transcriptional Regulators for Genes Dysregulated in DIO Zebrafish

**FIGURE 3:**
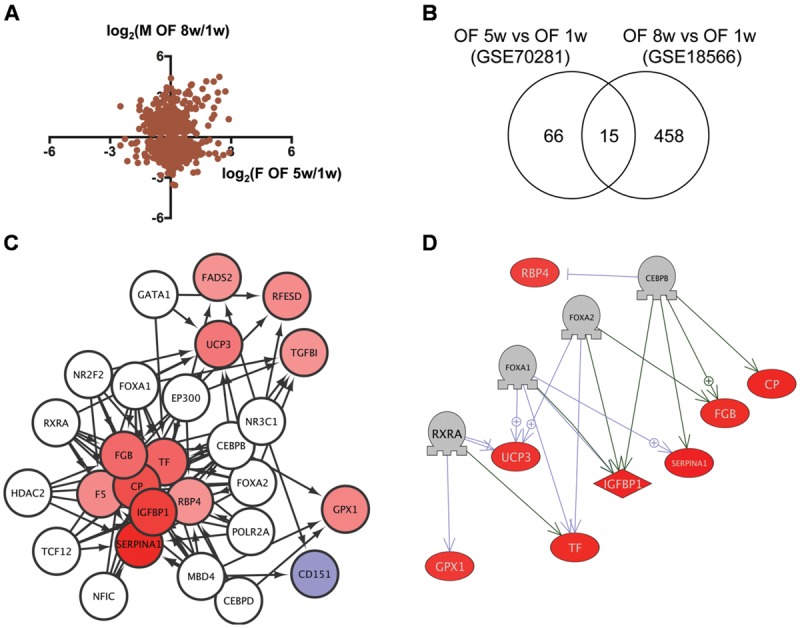
**Identification of the regulatory network for genes dysregulated in DIO zebrafish. (A)** Correlation of the change in gene expression in DIO female and male zebrafish. **(B)** Venn diagram of the numbers of DEG in DIO female and male zebrafish. **(C)** A regulatory network identified by iRegulon for the 15 common genes between female and male DIO zebrafish. Genes shown in blue and red indicate decreased and increased expression, respectively, by obesity. **(D)** A regulatory network identified by Pathway Studio for four regulators and their eight target genes. Genes shown in blue and red indicate decreased and increased expression, respectively, by obesity.

We then analyzed transcriptome data of visceral adipose tissues from female and male (Oka et al., 2010) DIO zebrafish to identify the network dysregulated in obesity. Although the correlation of gene expression between female and male DIO zebrafish was not high (**Figure 3A**), we identified 15 genes in common (**Figure 3B**, **Supplementary Table S1**, Sheet 3) between 81 and 473 genes dysregulated in female and male DIO zebrafish, respectively (**Supplementary Table S2**, Sheets 6 and 7). Gene ontology analysis revealed that 111 cell processes, including cell damage, superoxide anion generation and blood clotting, were enriched in the 15 genes identified (**Supplementary Table S3**, Sheet 3). iRegulon identified 14 transcriptional regulators for the 15 genes (**Supplementary Table S4**, Sheet 3). The network between these transcriptional regulators and their target genes is shown in **Figure 3C**. Pathway Studio identified 61 regulators for the 15 common genes (**Supplementary Table S5**, Sheet 3). Four regulators were overlapped between the iRegulon and Pathway Studio data. The network between the four regulators and their targets is shown in **Figure 3D**. The overlapping network correlated well with the data identified by iRegulon. Pathway Studio identified CEBPB as a positive regulator of *SERPINA1, CP, IGFBP1*, and *FGB*, all of which were also identified as targets of CEBPB by iRegulon (**Supplementary Table S4**, Sheet 3).

### Identification of Transcriptional Regulators for Genes Dysregulated in Obese Humans

**FIGURE 4:**
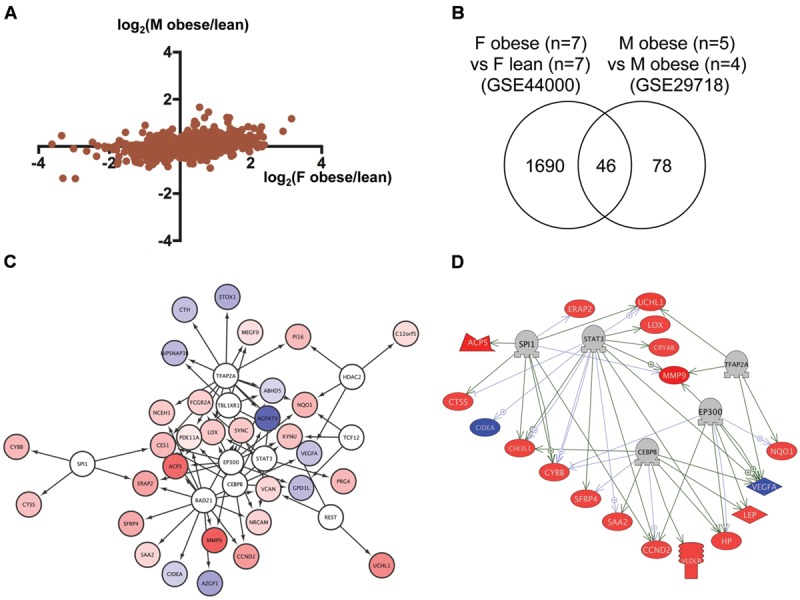
**Identification of the regulatory network for genes dysregulated in obese humans. (A)** Correlation of genes dysregulated in adipose tissues from female and male obese humans. **(B)** Venn diagram of the numbers of DEG in obese females and males. **(C)** A regulatory network identified by iRegulon for the 46 common genes between obese females and males. Genes shown in blue and red indicate decreased and increased expression, respectively, by obesity. **(D)** A regulatory network identified by Pathway Studio for five regulators and their 18 target genes. Genes shown in blue and red indicate decreased and increased expression, respectively, by obesity.

To identify regulatory network for genes dysregulated in obese humans, we analyzed the transcriptome data of adipose tissues from obese human females (Deng et al., 2013) and males (Tam et al., 2011) deposited to GEO as GSE44000 and GSE29718, respectively. As shown in **Figure 4A**, the correlation of gene expression between obese human females and males was relatively high (*r* = 0.37, *p* < 1 × 10^-4^). We identified 46 genes in common (**Figure 4B**; **Supplementary Table S1**, Sheet 4) between 1,736 and 124 genes dysregulated in obese human females and males, respectively (**Supplementary Table S2**, Sheets 8 and 9). Gene ontology analysis revealed that 350 cell processes, including lipid peroxidation, oxidative stress, and inflammatory response were enriched in the 46 genes (**Supplementary Table S3**, Sheet 4). Although there was no overlap between the 15 and 46 common genes in zebrafish and humans, respectively (**Supplementary Table S1**, Sheets 3 and 4), 73 gene ontologies were overlapped between the respective 111 and 350 gene ontologies in zebrafish and humans (**Supplementary Table S3**, Sheets 3 and 4). iRegulon identified 10 transcriptional regulators for the 46 common genes (**Supplementary Table S4**, Sheet 4). The network between these regulator and their targets is shown in **Figure 4C**. Pathway Studio identified 428 regulators for the 46 common genes (**Supplementary Table S5**, Sheet 4). Five regulators were overlapped between the iRegulon and Pathway Studio data. The regulatory network between the five regulators and their 18 target genes is shown in **Figure 4D**.

## Identification of Common Transcriptional Regulators Targeted by RSV and CR

**FIGURE 5:**
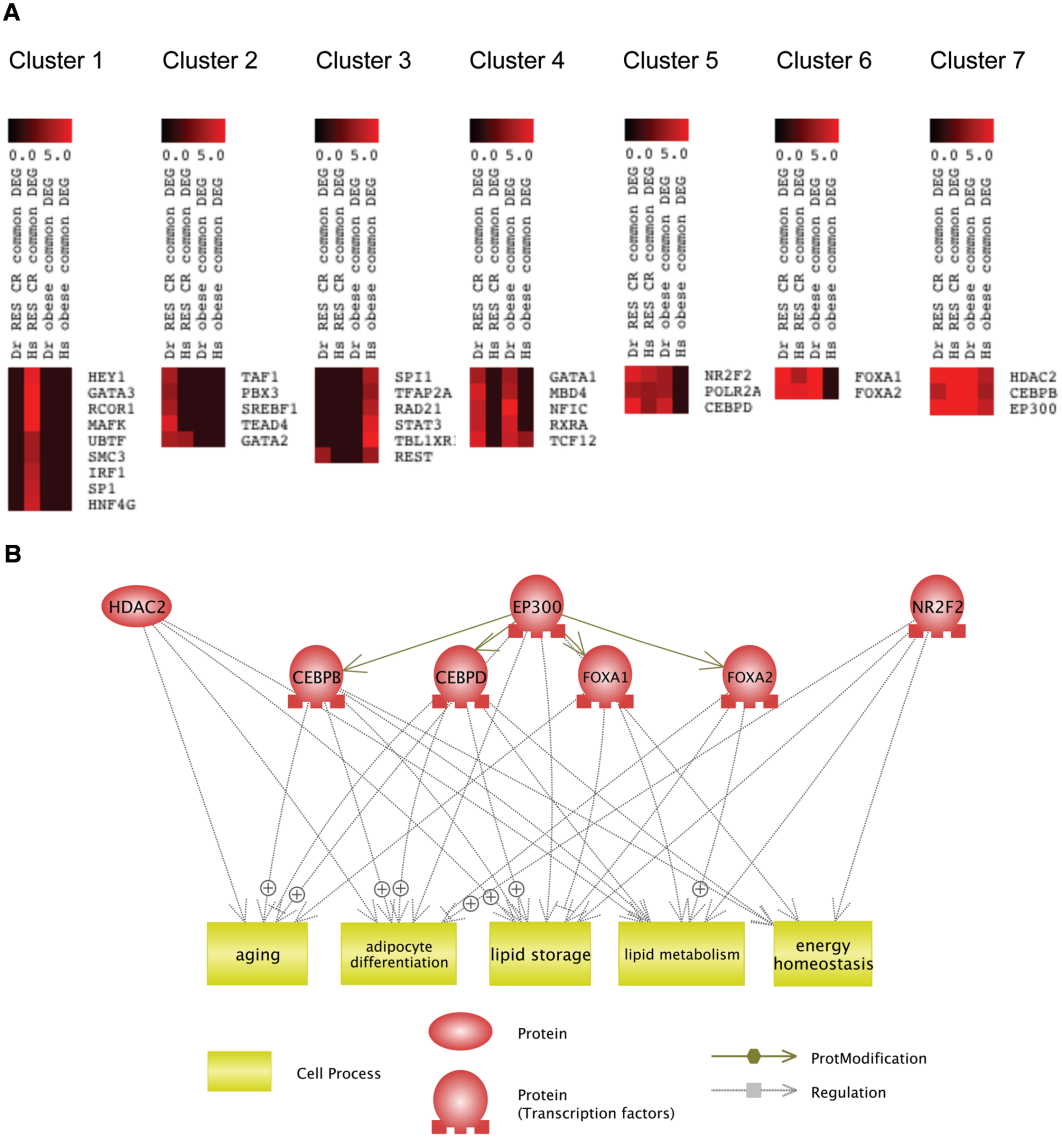
**Identification of common transcriptional regulators targeted by RSV and CR. (A)** *K*-Means clustering of regulators identified by iRegulon based on their NES in each analysis. **(B)** Network between transcriptional regulators in Clusters 5, 6, and 7 and cellular functions enriched in these transcriptional regulators and the genes dysregulated in obesity and normalized by RSV or CR.

To reveal which transcriptional regulators might be involved in the common mechanism of RSV and CR, we performed *K*-Means clustering (Soukas et al., 2000) of transcriptional regulators identified by iRegulon based on their NES (**Supplementary Table S4**). As shown in **Figure 5A**, the *K*-Means clustering classified these transcriptional regulators into seven clusters. The clustering revealed that HDAC2, CEBPB, and EP300 (Cluster 7) were transcriptional regulators for genes dysregulated in obesity and normalized by RSV and CR in both DIO zebrafish and obese human. NR2F2, POLR2A, CEBPD (Cluster 5), and FOXA1, FOXA2 (Cluster 6) were transcriptional regulators for genes dysregulated in DIO zebrafish and normalized by RSV and CR in both DIO zebrafish and obese humans. Gene ontology analysis revealed that 161 cell processes were enriched in the eight transcriptional regulators in Clusters 5, 6, and 7 (**Supplementary Table S3**, Sheet 5). Five cell processes (adipocyte differentiation, aging, energy homeostasis, lipid metabolism, and lipid storage) were overlapped with those enriched in genes dysregulated in obesity and normalized by RSV and CR in both DIO zebrafish and obese humans (**Supplementary Table S3**, Sheet 6). The network between these transcriptional regulators and cell processes identified by Pathway Studio is shown in **Figure 5B**. It was demonstrated that EP300 activates CEBPB, CEBPB, FOXA1, and FOXA2 by acetylation (Wang et al., 2006, 2013b; Cesena et al., 2007; Kohler and Cirillo, 2010). These findings suggest that EP300 might be a key transcriptional regulator involved in the common therapeutic mechanism of RSV and CR in adipose tissues of obese individuals.

### Inhibition of EP300 Reduces Adiposity in Larval Zebrafish

**FIGURE 6:**
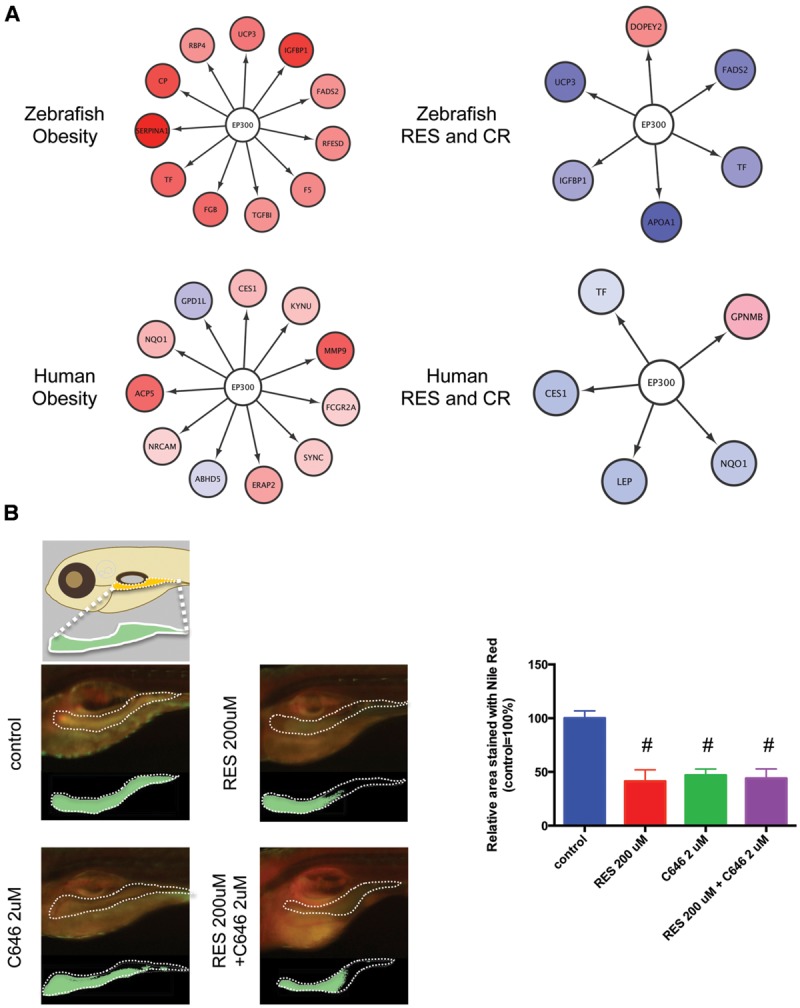
**Inhibition of EP300 reduces the adiposity in larval zebrafish. (A)** Network between EP300 and the target genes identified iRegulon in adipose tissues of DIO zebrafish and obese humans with and without RES and CR. **(B)** Changes in the visceral adiposity of zebrafish treated with RES and/or C646. The area stained with Nile Red is shown compared with that of control (arbitrarily defined as 100%). Values are the means ± SEM. *N* = 10–12/group. #*p* < 0.05 vs control.

The network between EP300 and the target genes identified by iRegulon is shown in **Figure 6A**. It was demonstrated that EP300 increased the expression of *TF* (Chaudhary and Skinner, 2001), *IGFBP1* (Nasrin et al., 2000), *NQO1* (Macoch et al., 2015), and *LEP* genes (Cascio et al., 2008). The expression of these genes increased in obesity (**Supplementary Figure S2**; **Supplementary Table S1**, Sheets 3 and 4) and was normalized by RSV and CR (**Supplementary Figure S2**; **Supplementary Table S1**, Sheets 1 and 2) in adipose tissues of zebrafish and humans. These results suggest that EP300 might be activated in obesity and that both RSV and CR may suppress the activity of EP300 in adipose tissues. To examine the effect of EP300 on adiposity, we used a selective EP300 inhibitor C646 that competes for AcCoA binding to EP300 (Bowers et al., 2010). As shown in **Figure 6B**, the abdominal area of larval zebrafish stained by Nile Red was significantly decreased by 2 μM C646, 200 μM RSV, or co-treatment of 2 μM C646 and 200 μM RSV. However, the reduction of adiposity was not significantly different among the three treatment groups. These results suggest that the inhibition of EP300 might be a major therapeutic mechanism of RSV. It was shown that CR decreased intracellular AcCoA (Marino et al., 2014) suggesting that CR might inhibit EP300 through reducing AcCoA.

## Discussion

In this study, we demonstrated that the inhibition of EP300 might be common therapeutic mechanism of CR and RSV in adipose tissues of obese individuals. EP300 is a transcriptional coactivator with various functions (Wang et al., 2013a), including linking DNA-bound TF to basal transcription machinery, relaxing chromatin structure through its histone acetyltransferase activity, and modulating the function of various proteins through the lysyl acetyltransferase activity (Wang et al., 2013a).

It was demonstrated that EP300 acetylated FXR, a master regulator of lipid homeostasis (Modica et al., 2010) and that the acetylation of FXR was constitutively activated in obesity (Kemper et al., 2009). EP300 also activated the transcription of genes encoding TG synthetic enzymes (Bricambert et al., 2010). Disrupting the function of EP300 in mice resulted in the reduction of white adipose tissue and plasma TG (Bedford et al., 2011). These findings suggest that EP300 might be activated in obesity and that the inhibition of EP300 would be a potential therapeutic strategy in obesity.

It was shown that RSV inhibited EP300 through the activation of SIRT1 (Shakibaei et al., 2011). When activated, SIRT1 promoted the deacetylation of lysine residues on EP300, resulting in the inhibition of acetyltransferase activity of EP300 (Kume et al., 2007). Because SIRT1 is also activated by CR (Guarente, 2013), CR might inhibit EP300 through the activation of SIRT1. However, CR also decreases intracellular AcCoA through SIRT3 (Hebert et al., 2013). Furthermore, CR inhibited EP300 through the depletion of AcCoA in the heart and skeletal muscle of mice, leading to the deacetylation of cellular proteins and activation of AMP-dependent protein kinase (Marino et al., 2014). These findings suggest that EP300 might be inhibited by RSV and CR in adipose tissues either dependently or independently of SIRT1.

In this study, we performed comparative transcriptome analysis of adipose tissues from DIO zebrafish and obese humans to identify a common therapeutic mechanism of RSV and CR. There are multiple methods to identify common pathways using comparative transcriptome analysis. The simplest way is to identify DEG that overlap among different transcriptome data. For example, we identified 46 DEG that overlapped between obese human females and males. Among the DEG, several genes were also identified as DEG in other transcriptome analyses not included in this study. These genes include carboxyl esterase 1 (*CES1*; Jernas et al., 2009), NAD(P)H dehydrogenase, quinone 1 (*NQO1*; Kim et al., 2011), cathepsin S (*CTSS*; Lee et al., 2005), matrix metallopeptidase 9 (*MMP9*; Lee et al., 2005). The expression of these genes was linked to adiposity (Taleb et al., 2005; Palming et al., 2007; Jernas et al., 2009), suggesting that the DEG replicated in multiple transcriptome data is a good and robust marker for obesity.

However, the lists of DEG do not always overlap between the same intervention in different strains/species or between related interventions in the same strains/species (Allison et al., 2006; Antosh et al., 2011). Therefore, the identification of overlapped DEG by comparative transcriptome analysis might be too stringent, resulting in overlooking important genes and pathways. To circumvent this, group testing is widely used. For example, the list of DEG from each transcriptome analysis can be compared with predefined gene sets related to specific functions calculating which gene sets are represented in the list of DEG at a level greater than that expected by chance alone (Manoli et al., 2006). By comparing the gene sets significantly enriched in each list of DEG, common pathways can be identified among multiple transcriptome data. Using this group analysis, several pathways have been successfully identified as common targets of CR and RSV, including “chromatin assembly or disassembly” (Barger et al., 2008). Functions related to chromatin were also identified as enriched categories in CR by another comparative transcriptome analysis (Wuttke et al., 2012). In this study, we performed *cis*-regulatory sequence analysis as a group analysis and identified EP300 as a common therapeutic target of CR and RSV. Taken together, these findings suggest that both CR and RSV may normalize the dysregulation of chromatin functions by EP300.

We also identified that the transcriptional regulators CEBPB and HDAC2 might be common therapeutic targets of RSV and CR. EP300 interacts with CEBPB at the promoter of targeted genes (Rastogi et al., 2013) and HDAC2 and EP300 compete for binding to promoters of their target genes (Meng et al., 2009). Indeed, the binding regions of EP300, CEBPB, and HDAC2 in the promoters of transferrin (*TF*; **Supplementary Figure S2**), insulin-like growth factor binding protein 1 (*IGFBP1*; **Supplementary Figure S3**) and *NQO1* (**Supplementary Figure S4**), whose expression was increased in obesity and was normalized by CR or RSV (**Figure 6A**), overlap. This might explain why iRegulon identified CEBPB and HDAC2 as transcriptional regulators. However, we cannot exclude the possibility that the activity of HDAC2 might be inhibited in obesity and normalized by CR and RSV. A previous study reported that mitogen-activated protein kinase phosphatase 3 deficient mice were protected from DIO and that the activation of HDAC2 by increased phosphorylation of Ser394 had a major role in the protection (Feng et al., 2014). Further studies are required to examine whether HDAC2 is involved in the therapeutic mechanism of CR and RSV.

## Conclusion

We demonstrated that the inhibition of EP300 might be a common therapeutic mechanism of CR and RSV. To our knowledge, this is the first study to indicate EP300 might be a potential therapeutic target of CR and RSV in adipose tissues. Chemicals and natural products that inhibit EP300 might function as CR mimetics to reduce adiposity in obesity.

## Author Contributions

YN conceived the study, analyzed the data and wrote the manuscript. SS performed experiments using larval zebrafish. MA, SI, and YS performed experiments using adult DIO zebrafish. KK and RK helped to perform the experiments. RY, TU, TY, and RT conceived the study and measured RSV contents in food. TT conceived the study and wrote the manuscript.

## Conflict of Interest Statement

The authors declare that the research was conducted in the absence of any commercial or financial relationships that could be construed as a potential conflict of interest.

## Supplementary Material

Figure S1 **Resveratrol reduces plasma triglyceride and visceral fat in DIO zebrafish. (A)** Changes in BMI (g/cm^2^) of zebrafish in the control, OF and OF + RSV groups. Values are means ± SEM, *N* = 6/group. **(B)** Changes in plasma TG levels of zebrafish in the control, OF and OF + RSV groups. Values are means ± SEM. Control: *N* = 19, OF: *N* = 14, OF + RSV: *N* = 15. **(C)** Changes in visceral adiposity of zebrafish in the control, OF and OF + RSV groups. Values are means ± SEM. *N* = 9/group. #*p* < 0.05 vs. control, ^∗^*p* < 0.05 vs. OF.

Figure S2 **Binding of transcriptional regulators to the human *TF* gene.** These data were based on the UCSC Genome Browser. The binding of EP300, HDAC2, and CEBPB are shown as red, blue, or green arrows.

Figure S3 **Binding of transcriptional regulators to the human *IGFBP1* gene.** These data were based on the UCSC Genome Browser. The binding of EP300, HDAC2, and CEBPB are shown as red, blue, or green arrows.

Figure S4 **Binding of transcriptional regulators to the human *NQO1* gene.** These data were based on the UCSC Genome Browser. The binding of EP300, HDAC2, and CEBPB are shown as red, blue, or green arrows.

Table S1 **Genes with changes in expression are common between multiple datasets.** Sheet 1: Genes with changes in expression are common between RSV and CR in visceral adipose tissue in DIO zebrafish. Sheet 2: Genes with changes in expression are between RSV and CR in subcutaneous adipose tissues of obese humans. Sheet 3: Genes dysregulated in visceral adipose tissues of both female and male DIO zebrafish. Sheet 4: Genes dysregulated in subcutaneous adipose tissues of obese human females and males.

Table S2 **Differentially expressed genes in each transcriptome dataset.** Sheet 1: Genes regulated by RSV in visceral adipose tissues of female DIO zebrafish. Sheet 2: Genes regulated by CR in visceral adipose tissues of male DIO zebrafish. Sheet 3: Genes regulated by RSV in subcutaneous adipose tissues from obese human males treated with RSV. Sheet 4: Genes regulated by CR in subcutaneous adipose tissues from obese human females. Sheet 5: Genes regulated by CR in subcutaneous adipose tissues from obese human males. Sheet 6: Genes dysregulated in visceral adipose tissues of female DIO zebrafish. Sheet 7: Genes dysregulated in visceral adipose tissues of male DIO zebrafish. Sheet 8: Genes dysregulated in subcutaneous adipose tissues of obese human females. Sheet 9: Genes dysregulated in subcutaneous adipose tissues of obese human males.

Table S3 **Cell processes enriched in the gene list.** Sheet 1: Cell processes enriched in the common genes between RSV and CR in visceral adipose tissues of DIO zebrafish. Sheet 2: Cell processes enriched in the common genes between RSV and CR in subcutaneous adipose tissues of obese humans. Sheet 3: Cell processes enriched in the common genes dysregulated in visceral adipose tissues of female and male DIO zebrafish. Sheet 4: Cell processes enriched in the common genes dysregulated in subcutaneous adipose tissues of obese human females and males. Sheet 5: Cell processes enriched in HDAC2, EP300, CEBPB, CEBPD, FOXA1, FOXA2, NR2F2, and POLR2A. Sheet 6: Common cell processes enriched in genes between RSV and CR, in genes between obese females and males, and in eight regulators.

Table S4 **Transcriptional regulators identified by iRegulon.** Sheet 1: Transcriptional regulators identified by iRegulon for the common genes between RSV and CR in visceral adipose tissues of DIO zebrafish. Sheet 2: Transcriptional regulators identified by iRegulon for the common genes between RSV and CR in subcutaneous adipose tissues of obese humans. Sheet 3: Transcriptional regulators identified by iRegulon for genes dysregulated in common between female and male DIO zebrafish. Sheet 4: Transcriptional regulators identified by iRegulon for genes dysregulated in common between obese human females and males.

Table S5 **Transcriptional regulators identified by Pathway Studio.** Sheet 1: Transcriptional regulators identified by Pathway Studio for the common genes between RSV and CR in visceral adipose tissues of DIO zebrafish. Sheet 2: Transcriptional regulators identified by Pathway Studio for the common genes between RSV and CR in subcutaneous adipose tissues of obese humans. Sheet 3: Transcriptional regulators identified by Pathway Studio for the common genes between female and male DIO zebrafish. Sheet 4: Transcriptional regulators identified by Pathway Studio for the common genes between female and male DIO zebrafish.

## Abbreviations

AcCoA: acetyl coenzyme A
BMI: body mass index
CR: caloric restriction
DIO: diet-induced obese
dpf: days post-fertilization
FXR: farnesoid X receptor
GEO: Gene Expression Omnibus
mpf: months post-fertilization
OF: overfeeding
RSV: resveratrol
TF: transcription factors
TGs: triglycerides
