# Supplementary figures and images for: Systems pharmacology of adiposity reveals inhibition of EP300 as a common therapeutic mechanism of caloric restriction and resveratrol for obesity
Source: Front Pharmacol. 2015 Sep 15;6:199. doi: 10.3389/fphar.2015.00199 (PMC4569862; doi:10.3389/fphar.2015.00199)

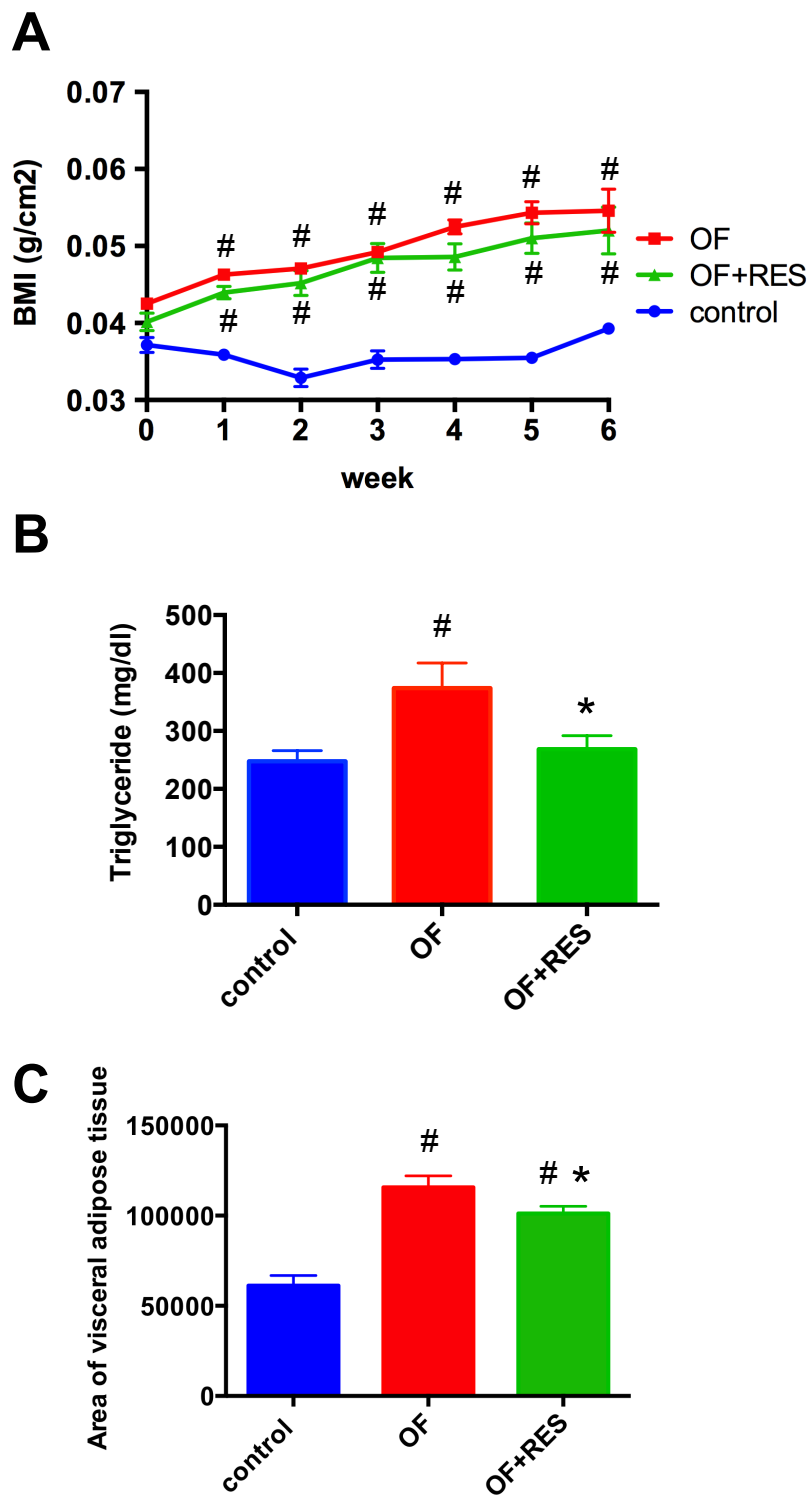

Figure S1. Resveratrol reduces plasma triglyceride and visceral fat in DIO zebrafish.

Supplement: Figure S1 — Resveratrol reduces plasma triglyceride and visceral fat in DIO zebrafish. (A) Changes in BMI (g/cm2) of zebrafish in the control, OF and OF + RSV groups. Values are means ± SEM, N = 6/group. (B) Changes in plasma TG levels of zebrafish in the control, OF and OF + RSV groups. Values are means ± SEM. Control: N = 19, OF: N = 14, OF + RSV: N = 15. (C) Changes in visceral adiposity of zebrafish in the control, OF and OF + RSV groups. Values are means ± SEM. N = 9/group. #p < 0.05 vs. control, ∗p < 0.05 vs. OF. [file Image_1.PDF]
