# Supplementary material for: Systems pharmacology of adiposity reveals inhibition of EP300 as a common therapeutic mechanism of caloric restriction and resveratrol for obesity
Source: Front Pharmacol. 2015 Sep 15;6:199. doi: 10.3389/fphar.2015.00199 (PMC4569862; doi:10.3389/fphar.2015.00199)
Supplement: Figure S2 — Binding of transcriptional regulators to the human TF gene. These data were based on the UCSC Genome Browser. The binding of EP300, HDAC2, and CEBPB are shown as red, blue, or green arrows. [file Image_2.PDF]

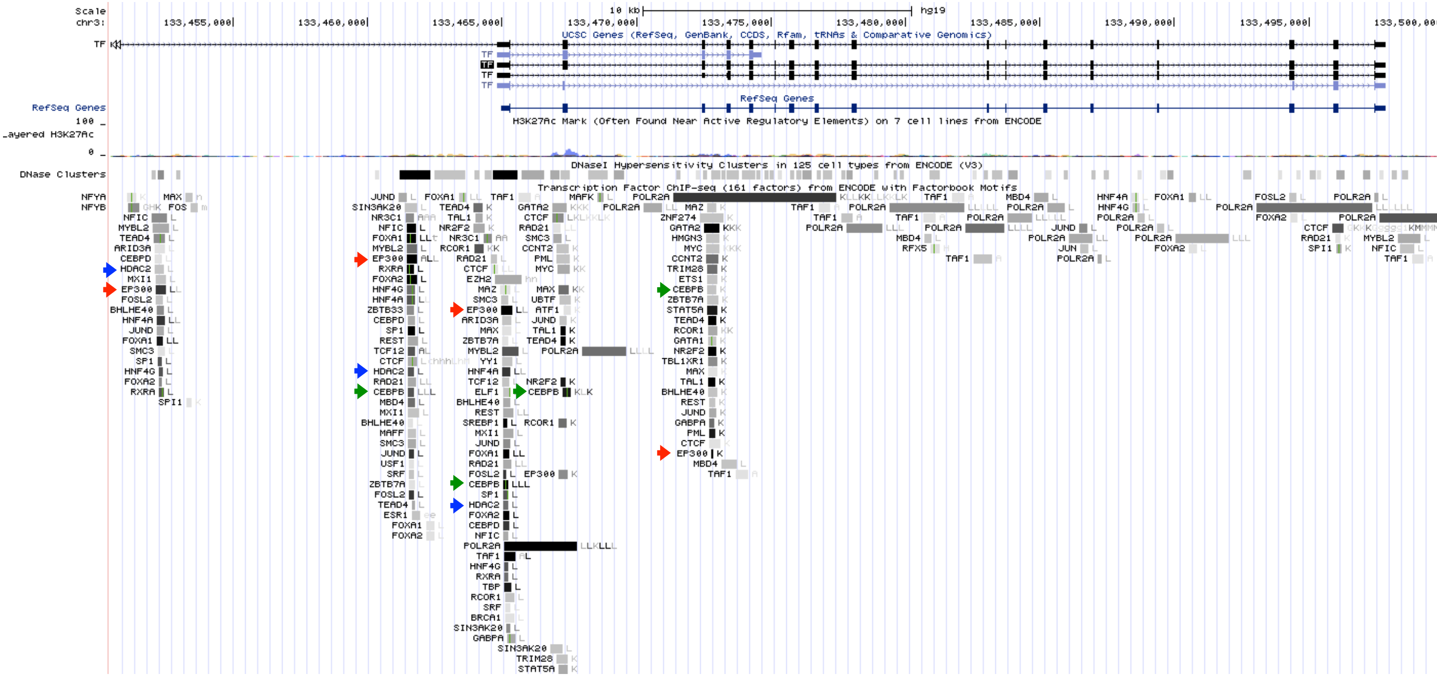

Figure S2. Binding of transcriptional regulators to the human TF gene.
